# Secondary cytoreductive surgery and hyperthermic intraperitoneal chemotherapy for recurrent colorectal peritoneal metastases

**DOI:** 10.1016/j.sopen.2024.05.018

**Published:** 2024-05-31

**Authors:** Peter Harald Cashin, Dan Asplund, Elinor Bexe Lindskog, Lana Ghanipour, Ingvar Syk, Wilhelm Graf, Per J. Nilsson, Gabriella Jansson Palmer

**Affiliations:** a Uppsala University Hospital, Uppsala, Sweden; b Region Västra Götaland, Sahlgrenska University Hospital, Dept of Surgery, Gothenburg, Sweden; c Skåne's University Hospital, Malmö, Sweden; d Karolinska University Hospital & Karolinska Institutet, Stockholm, Sweden; e Department of Surgery, Institute of Clinical Sciences, Sahlgrenska Academy, University of Gothenburg, Gothenburg, Sweden

**Keywords:** Colorectal cancer, Peritoneal metastases, Cytoreductive surgery, Hyperthermic intraperitoneal chemotherapy, Relapse treatment, Systemic chemotherapy

## Abstract

**Background:**

Secondary treatment of recurrent colorectal peritoneal metastases after previous cytoreductive surgery (CRS) and hyperthermic intraperitoneal chemotherapy (HIPEC) is poorly investigated.

**Objectives:**

To evaluate the overall survival outcome of secondary (repeat) CRS + HIPEC compared to palliative treatment in recurrent peritoneal disease.

**Methods:**

Patients with colorectal peritoneal metastases treated with an index CRS + HIPEC and subsequently having recurrent peritoneal disease were identified from the prospective Swedish national HIPEC registry. Patients were divided into interventional group (secondary CRS + HIPEC) or palliative group. Multivariable logistic regression, propensity-score matching, and survival outcomes were calculated.

**Results:**

Among 575 patients who underwent complete CRS between 2010 and 2021, 208 (36 %) were diagnosed with a subsequent recurrent peritoneal disease. Forty-two patients (20 %) were offered secondary CRS + HIPEC. Propensity-score matching of secondary interventional cases with palliative cases succeeded in 88 % (*n* = 37) in which female sex, lower peritoneal cancer index at index surgery, longer disease-free interval, and absence of extra-peritoneal metastases were identified as the most relevant matching covariates. Median OS from date of recurrence was 38 months (95%CI 30–58) in the interventional group and 19 months (95%CI: 15–24) in the palliative group (HR 0.35 95%CI: 0.20–0.63, *p* = 0.0004). Sensitivity analyses confirmed the results. As reference, the median OS from index CRS + HIPEC in the whole colorectal registry (*n* = 575) was 41 months (95%CI: 38–45).

**Conclusion:**

After matching for relevant factors, the hazard ratio for death was significantly reduced in patients who were offered a secondary CRS + HIPEC procedure for recurrent peritoneal disease. Selection bias is inherent, but survival outcomes were comparable to those achieved after the initial procedure.

## Introduction

Cytoreductive surgery (CRS) with hyperthermic intraperitoneal chemotherapy (HIPEC) is a standard treatment option for patients with resectable colorectal peritoneal metastases (PM), although the HIPEC component is under some scrutiny [1]. A recent trial with mitomycin C in the prophylactic setting has suggested a locoregional effect on the peritoneum [2]. Regardless of the use of HIPEC, CRS is a treatment cornerstone for resectable colorectal PM [3,4].

It is still unclear whether a second CRS and HIPEC treatment after peritoneal recurrence following initial CRS and HIPEC provides any survival advantage over systemic chemotherapy. There are previous studies evaluating repeat CRS and HIPEC, but without any relevant comparative group [[5], [6], [7], [8], [9], [10], [11], [12], [13]]. Selecting patients for a secondary procedure is a difficult decision. Many factors must be taken into account in this situation. It may be that the patients chosen for a second procedure would have done just as well on systemic chemotherapy, but any relevant comparison groups are missing in these studies except for one [7].

The aim of this study was to evaluate the outcome of secondary (first repeat) CRS and HIPEC in patients who had already undergone the treatment once. The primary outcome was overall survival. Compared between the group of patients who were offered secondary surgery with HIPEC and the group who went for palliative treatment only. This was a Swedish Peritoneal Oncology Group study.

## Materials and methods

All patients in the Swedish HIPEC Registry who underwent complete CRS and HIPEC due to colorectal PM between 2010 and 2021 were extracted. From this group, patients who had a subsequent peritoneal recurrence were selected for the decision-making analysis, i.e. analysis to identify predictive factors related to receiving secondary CRS. Whether a patient was offered secondary CRS and HIPEC or not was modelled by uni- and multivariable logistic regression with secondary intervention as endpoint, where baseline variables that differed between groups were included. For prognostic evaluation, a univariate Cox regression analysis on overall survival was also performed on these baseline variables. Variables with a *p*-value <0.1 in the logistic regression analysis and variables considered prognostically important in the Cox regression (*p* < 0.1) were defined as relevant criteria in the decision-making analysis. Subsequently, the following variables were included in the propensity score matching process: sex, peritoneal cancer index (PCI) at index CRS and HIPEC, disease-free interval, and the absence of extra-peritoneal metastases. Operating time was also statistically significant, but due to significant collinearity between PCI and operating time, the PCI was chosen instead. This study was approved by the Ethical Review Authority (Dnr 2023–03743-01).

### Treatments

The current Swedish national standard HIPEC regime for colorectal peritoneal metastases is the 30-min oxaliplatin-based HIPEC (460 mg/m^2^) with bidirectional 5-fluorouracil intravenously (400 mg/m^2^). Currently, in Sweden, a RCT is underway comparing this standard with an intensified HIPEC treatment regime due to the results of the PRODIGE 7 study [1]. 87 % (*n* = 180) received of patients in this cohort received the standard HIPEC treatment. 10 % (*n* = 21) received an irinotecan-based HIPEC (360-400 mg/m^2^) with bidirectional intravenous 5-fluorouracil (400 mg/m^2^). The remaining 3 % (*n* = 6) received mitomycin C HIPEC (30–35 mg/m^2^).

### Statistics

The process of choosing the variables for propensity score matching (PSM) is described above. The nearest neighbor method without replacement and with a caliper of 0.1 was used to define the propensity score matching process. Both the recurrent cohort (*n* = 208) and the PSM cohort (*n* = 74) was compared with descriptive statistics. The overall survival (OS) was compared between groups using Kaplan-Meier curves and log-rank test. Cox regression analysis was used to calculate hazard ratios. Signet-cell histology was not included in Cox regression due to few cases in the interventional group. A *p*-value of <0.05 was defined as statistically significant.

### Sensitivity analyses statistics

Several sensitivity analyses were performed. First, an unadjusted Kaplan-Meier curve in the recurrent cohort was rendered. Second, a multivariable Cox regression analyses using the most often identified prognostic factors from the literature was performed for the recurrent cohort. Third, the same multivariable Cox regression was performed for the propensity score matched cohort. Fourth, several propensity-score matched methods were tested to investigate whether propensity score methodology affected the results. Lastly, an analysis of survival from index surgery was performed in the propensity score matched cohort.

## Results

**Fig. 1:**
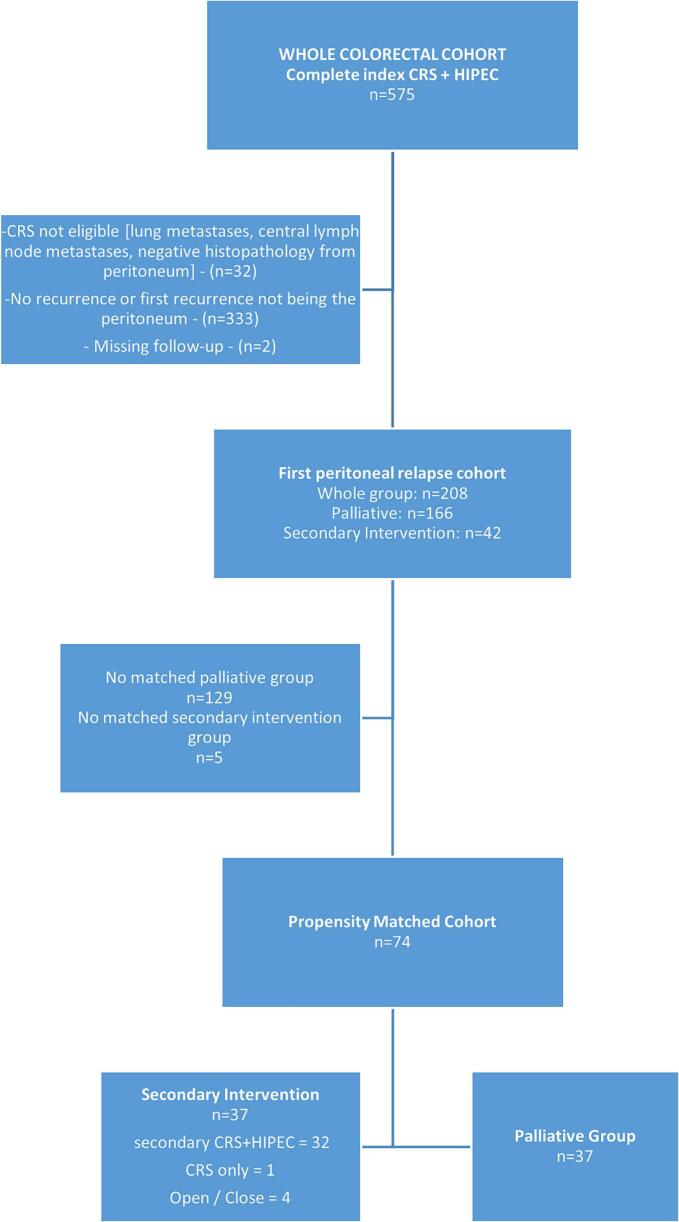
Flowchart – Swedish HIPEC Registry 2010–2021.

**Fig. 2:**
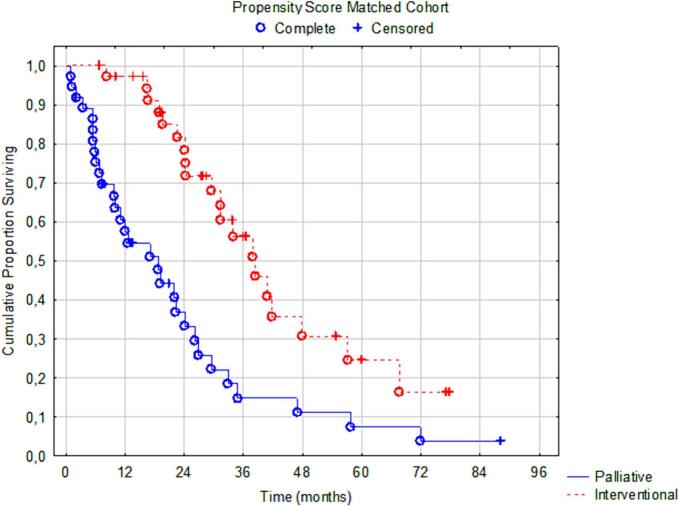
Overall survival in the propensity score matched cohort (n = 74) from date of first relapse to death – *p* = 0.00038. Median OS 38 vs 19 months.

**Fig. S1:**
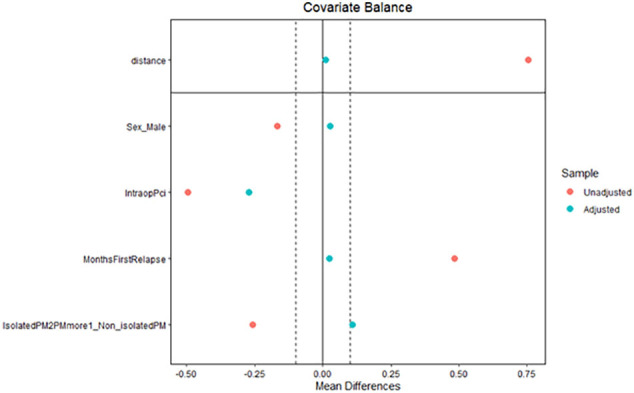
Love plot of the propensity score matching.

**Fig. S2:**
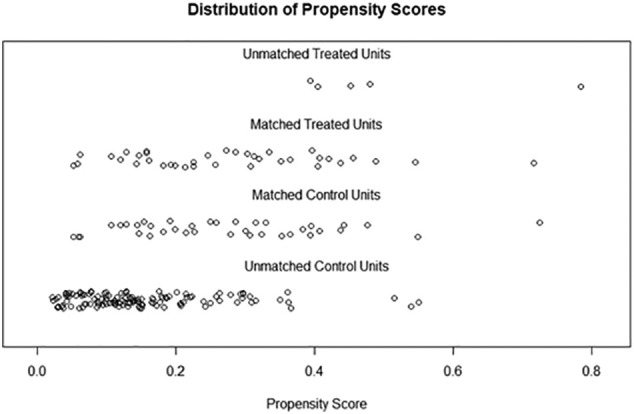
Jitter plot distribution of propensity scores.

Among 575 patients who underwent complete CRS for colorectal peritoneal metastases 2010–2021 (Fig. 1), 208 (36 %) were diagnosed with a peritoneal recurrence – the recurrent cohort (Fig. 1). In Table 1, the patient and tumor characteristics and treatment are described in the two groups. Forty-two patients (20 %) were offered secondary CRS + HIPEC, whereas 166 patients were selected for palliative chemotherapy or best supportive care. Female sex, PCI at index surgery, disease-free interval, and extra-peritoneal metastases were identified as relevant matching factors according to the relevancy criteria described in the methods section (Table 2). The baseline factors were well-balanced in the propensity score matched cohort except for four factors (age, tumor sidedness, neoadjuvant therapy, operating time), Table 3. Propensity-score matching using the variables above successfully matched 37 cases in the interventional group with 37 palliative cases (Supplementary Fig. S1, Fig. S2). OS in the matched cohort from date of peritoneal recurrence was 38 months (95%CI 30–58) in the secondary intervention group and 19 months (95%CI: 15–24) in the palliative group (HR 0.35 95%CI: 0.20–0.63, *p* = 0.0004, Fig. 2). The median OS from index surgery in the whole cohort (*n* = 575) was 41 months (95%CI: 38–45). Four patients in the interventional group were unable to receive the planned intervention (Table 3).

### Sensitivity analyses results

**Fig. 3:**
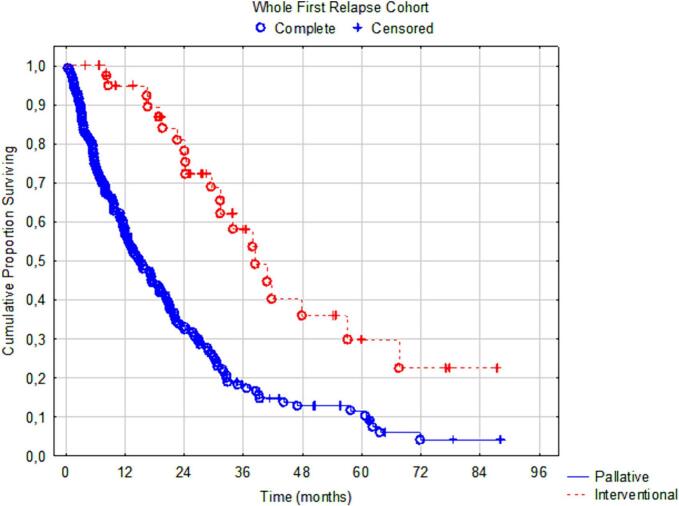
Overall survival in the first relapse cohort (*n* = 208) from date of first relapse to death – *p* < 0.00001. Median OS 38 vs 15 months.

**Fig. S3:**
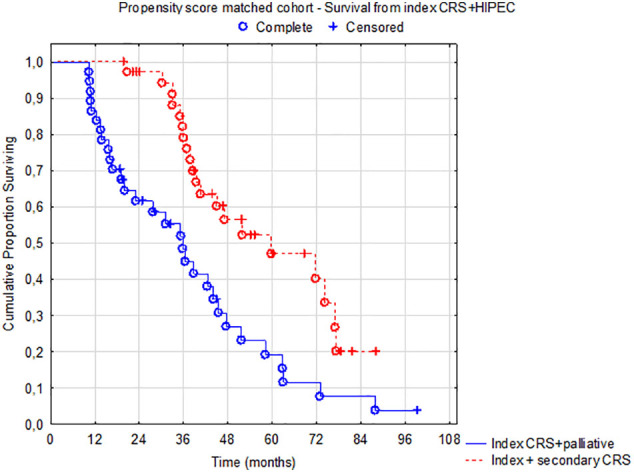
Overall survival from index surgery to death for the propensity score matched group (*n* = 74, *p* = 0.002).

Fig. 3 shows a median OS of 38 month (95%CI: 29–68) in the interventional group in the unmatched recurrent cohort (*n* = 208) compared to 15 months (95%CI: 13–20) in the palliative group. The multivariable HR for death in the interventional group was 0.29 (95%CI: 0.16–0.54, *p* < 0.0001) in the unmatched recurrent cohort and was 0.23 (95%CI: 0.10–0.52, *p* = 0.0004) for the propensity score matched cohort (Table 4). Regardless of propensity score methodology, no relevant changes in outcomes were noted (data not shown). In the propensity score matched cohort, median OS from index surgery was 60 months (95%CI: 44–77) in the interventional group vs 36 months (95%CI: 31–42) in the palliative group (HR 0.40 95%CI: 0.23–0.71, *p* = 0.0017), Fig. S3.

**Table 1:** – First peritoneal relapse cohort (n = 208).

	Palliative *n* = 166	Interventional *n* = 42	p	SMD
**Demography & tumor characteristics**				
Female – n (%)	83 (50.0)	28 (66.7)	0.078	0.343
Male – n (%)	83 (50.0)	14 (33.3)		
Metachronous – n (%)	71 (42.8)	16 (38.1)	0.709	0.095
Synchronous – n (%)	95 (57.2)	26 (61.9)		
Right-sided primary – n (%)	106 (63.9)	18 (42.9)	0.029	0.454
Left-sided primary – n (%)	42 (25.3)	19 (45.2)		
Rectum primary – n (%)	18 (10.8)	5 (11.9)		
T-stage Missing Data – n (%)	12 (7.3)	1 (2.4)	0.472	0.418
T2 – n (%)	3 (1.8)	2 (4.9)		
T3 – n (%)	37 (22.4)	8 (19.5)		
T4a – n (%)	70 (42.4)	16 (39.0)		
T4b – n (%)	43 (26.1)	14 (34.1)		
N-stage Missing Data – n (%)	12 (6.2)	1 (2.4)	0.119	0.519
N0 – n (%)	16 (9.6)	10 (23.8)		
N1 – n (%)	60 (36.1)	17 (40.5)		
N2 – n (%)	75 (45.2)	14 (33.3)		
NX – n (%)	3 (1.8)	0 (0.0)		
Signet-ring cells Missing Data – n (%)	32 (19.3))	6 (14.3)	0.420	0.330
No – n (%)	116 (69.9)	34 (81.0)		
Yes – n (%)	18 (10.8)	2 (4.8)		
**Variables at Index CRS** **+** **HIPEC**				
Age (range 26–79) – mean years (SD)	62.45 (9.71)	59.02 (11.63)	0.052	0.319
CEA – mean (SD)	61.11 (218.24)	40.00 (101.47)	0.554	0.124
CA19–9 – mean (SD)	109.82 (228.01)	58.10 (138.56)	0.181	0.274
CA 125 – mean (SD)	48.38 (78.70)	42.59 (100.13)	0.707	0.064
Neoadjuvant chemotherapy – n (%)	30 (18.1)	12 (28.6)	0.461	0.276
Adjuvant chemotherapy – n (%)	91 (54.8)	23 (54.8)	0.501	0.275
Operating time at index CRS – mean min (SD)	519.64 (150.30)	426.07 (135.59)	<0.001	0.654
Index PCI – mean (SD)	13.54 (6.76)	10.93 (5.27)	0.021	0.430
Liver metastases removed at index CRS – n (%)	13 (11.8)	4 (12.1)	1.000	0.009
Neutropenia after index CRS – n (%)	12 (7.2)	2 (4.8)	0.847	0.105
Clavien-Dindo >3 after index CRS – n (%)	47 (28.3)	9 (21.4)	0.143	0.641
IIIa – n (%)	22 (13.3)	7 (17.1)		
IIIb – n (%)	21 (12.7)	1 (2.4)		
IVa – n (%)	2 (1.2)	1 (2.4)		
IVb – n (%)	2 (1.2)	0 (0.0)		
Length of stay in days – mean (SD)	17 (17)	12 (6)	0.073	0.333
90-day mortality from 1:st relapse – n (%)	14 (8.4)	0 (0.0)	<0.001	0.788
**Variables at First Peritoneal Relapse**				
Isolated peritoneal relapse – n (%)	68 (41.0)	28 (66.7)	0.005	0.534
Peritoneal + other site relapse – n (%)	98 (59.0)	14 (33.3)		
Time Index to First Relapse – mean months (SD)	11.74 (7.99)	16.91 (10.68)	0.001	0.549

SMD – standardized mean difference, CEA – carcinoembryonic antigen, CA – cancer antigen, CRS – cytoreductive surgery, HIPEC – hyperthermic intraperitoneal chemotherapy, PCI – peritoneal cancer index, SD – standard deviation.

**Table 2:** Uni and Multivariable Logistic Regression and Cox regression – Decision-making analysis on recurrence cohort (n = 208). Results of OR > 1 meant more likely palliative group while OR < 1 meant more likely offered secondary CRS + HIPEC. HR is calculated from the date of recurrence to death of any cause. A *p*-value of at least <0.1 in all three categories were required to be included as factor in propensity score matching.

Variable	Uni OR	p	Multi OR	p	HR uni	p
Female	0.50 (0.25–1.02)	0.056	0.50 (0.22–1.12)	0.091	0.70 (0.51–0.96)	0.026
Male	Reference					
Age at index surgery	1.03 (1.00–1.06)	0.055	1.04 (1.00–1.07)	0.044	1.00 (0.99–1.02)	0.567
Operating time (min)	1.005 (1.002–1.008)	0.0005	NA⁎		NA⁎	
PCI at index surgery	1.07 (1.01–1.13)	0.023	1.08 (1.01–1.15)	0.016	1.03 (1.01–1.06)	0.0091
Right-sided Primary	1.64 (0.54–5.96)	0.385	1.48 (0.43–5.11)	0.539	1.16 (0.71–1.89)	0.214
Left-sided Primary	0.61 (0.20–1.90)	0.397	0.40 (0.11–1.43)	0.158	0.88 (0.51–1.49)	0.296
Rectum	Reference					
Months to First Relapse	0.94 (0.91–0.98)	0.0013	0.94 (0.90–0.98)	0.0022	0.96 (0.94–0.99)	0.0012
Isolated First Peritoneal Relapse	Reference					
Non-isolated First Peritoneal Relapse	2.88 (1.41–5.86)	0.0036	2.84 (1.27–6.35)	0.011	1.33 (0.96–1.81)	0.084

OR – Odds ratio, HR – hazard ratio, PCI – peritoneal cancer index.

⁎ Due to collinearity between PCI and operating time, the PCI was chosen as the more prognostically important factor.

**Table 3:** Propensity matched cohort (n = 74).

	Palliative n = 37	Interventional *n* = 37	p	SMD
**Demography & tumor characteristics**				
Female – n (%)	26 (70.3)	25 (67.6)	1.000	0.058
Male – n (%)	11 (29.7)	12 (32.4)		
Liver metastases – n (%)	1 (4.0)	4 (13.8)	0.443	0.349
Metachronous – n (%)	17 (45.9)	13 (35.1)	0.478	0.222
Synchronous – n (%)	20 (54.1)	24 (64.9)		
Right-sided primary – n (%)	26 (70.3)	15 (40.5)	0.037	0.627
Left-sided primary – n (%)	9 (24.3)	18 (48.6)		
Rectum primary – n (%)	2 (5.4)	4 (10.8)		
T-stage Missing Data – n (%)	5 (13.5)	1 (2.8)	0.481	0.515
T2 – n (%)	1 (2.7)	1 (2.8)		
T3 – n (%)	7 (18.9)	6 (16.7)		
T4a – n (%)	14 (37.8)	15 (41.7)		
T4b – n (%)	10 (27.0)	13 (36.1)		
N-stage Missing Data – n (%)	5 (13.5)	1 (2.8)	0.230	0.573
N0 – n (%)	2 (5.4)	7 (18.9)		
N1 – n (%)	16 (43.2)	15 (40.5)		
N2 – n (%)	14 (37.8)	14 (37.8)		
Signet-ring cells Missing Data – n (%)	10 (27.0)	5 (13.5)	0.156	0.551
No – n (%)	24 (64.9)	31 (83.8)		
Yes – n (%)	3 (8.1)	1 (2.7)		
**Variables at Index CRS** **+** **HIPEC**				
Age – mean years (SD)	63.65 (8.06)	58.73 (12.08)	0.043	0.479
CEA – mean (SD)	34.90 (74.01)	30.26 (70.05)	0.794	0.064
CA19–9 – mean (SD)	88.32 (188.40)	64.35 (147.53)	0.576	0.142
CA 125 – mean (SD)	32.79 (38.46)	45.56 (107.11)	0.551	0.159
Neoadjuvant chemotherapy – n (%)	3 (8.1)	11 (29.7)	0.050	0.593
Adjuvant chemotherapy – n (%)	17 (45.9)	20 (54.1)	0.609	0.318
Operating time at index CRS – mean min (SD)	505.57 (132.77)	425.46 (144.24)	0.015	0.578
Index PCI – mean (SD)	12.65 (6.75)	11.22 (5.44)	0.319	0.234
Neutropenia after index CRS – n (%)	5 (13.5)	1 (2.7)	0.202	0.425
Clavien-Dindo >3 after index CRS – n (%)	9 (24.3)	8 (21.6)	0.556	0.592
IIIa – n (%)	3 (8.1)	6 (16.7)		
IIIb – n (%)	4 (10.8)	1 (2.8)		
IVa – n (%)	1 (2.7)	1 (2.8)		
IVb – n (%)	1 (2.7)	0 (0.0)		
Length of stay in days – mean (SD)	18 (18)	12 (5)	0.061	0.303
90-day mortality from 1:st relapse date – n (%)	4 (10.8)	0 (0.0)	0.115	0.510
**Variables at First Peritoneal Relapse**				
Isolated peritoneal relapse – n (%)	27 (73.0)	23 (62.2)	0.456	0.232
Peritoneal + other site relapse – n (%)	10 (27.0)	14 (37.8)		
Time Index to First Relapse – mean months (SD)	15.01 (10.94)	15.26 (9.41)	0.919	0.024
**Variables at planned Secondary Intervention**				
Open/close with no secondary intervention n (%)	NA	4 (11)		
PCI – mean (SD)	NA	8.55 (5.69)		
PCI – missing data n (%)	NA	3 (8)		
Delta PCI compared to index PCI – mean (SD)	NA	−1.43 (7.11)		
CC score 0 of secondary CRS (*n* = 33) – n (%)⁎	NA	31 (94)		
Switched HIPEC regimen (*n* = 32) – n (%)	NA	16 (50)		
No HIPEC regimen switch (n = 32) – n (%)	NA	5 (16)		
Missing data on switch (n = 32) – n (%)	NA	11 (34)		
Clavien-Dindo >3 after CRS (n = 33) – n (%)	NA	6 (18)		
Length of stay in days – mean (SD)	NA	12 (9)		

SMD – standardized mean difference, CEA – carcinoembryonic antigen, CA – cancer antigen, CRS – cytoreductive surgery, HIPEC – hyperthermic intraperitoneal chemotherapy, PCI – peritoneal cancer index, SD – standard deviation, CC score – completeness of cytoreduction.

⁎ One patient underwent CRS without HIPEC.

**Table 4:** Multivariable Cox regression analysis from date of first relapse to endpoint death.

Variable	Multi HR – whole cohort	P	Multi - HR PSM cohort	p
Interventional vs palliative	0.29 (0.16–0.54)	<0.0001	0.23 (0.10–0.52)	0.0004
Female	0.58 (0.38–0.91)	0.017	1.04 (0.48–2.24)	0.915
Male	Reference		Reference	
Age	0.99 (0.97–1.01)	0.499	0.98 (0.94–1.02)	0.418
Liver metastases	1.28 (0.69–2.39)	0.434	5.60 (1.16–21.1)	0.032
Index PCI	1.01 (0.98–1.04)	0.474	1.03 (0.98–1.09)	0.283
Right-sided Primary	0.83 (0.46–1.51)	0.544	1.05 (0.29–3.77)	0.938
Left-sided Primary	0.54 (0.27–1.07)	0.077	0.40 (0.12–1.32)	0.130
Rectum Primary	Reference		Reference	
Lymph-node positive	1.37 (0.70–2.68)	0.361	1.46 (0.42–5.09)	0.555
Lymph-node negative	Reference		Reference	
Missing data Lymph-node	1.58 (0.65–3.83)	0.431	9.04 (1.69–48.4)	0.010
Adjuvant Chemotherapy	0.69 (0.43–1.11)	0.129	0.42 (0.17–1.04)	0.061
No Adjuvant Chemotherapy	Reference		Reference	
Missing data adjuvant	0.21 (0.03–1.67)	0.141	1.01 (0.20–5.17)	0.989
Months to First Relapse	0.98 (0.96–1.01)	0.183	0.99 (0.96–1.03)	0.702
Isolated First Peritoneal Relapse	Reference		Reference	
Non-isolated First Peritoneal Relapse	0.81 (0.52–1.26)	0.346	0.39 (0.16–0.95)	0.037

HR – hazard ratio, PSM – propensity score matched, PCI – peritoneal cancer index.

## Discussion

In this national, population-based, prospectively registered cohort, the intention to treat a peritoneal recurrence with a secondary CRS and HIPEC (interventional group) was associated with better survival than palliative treatment intent (palliative group) in the unmatched recurrence study cohort (*n* = 208), 38 vs. 15 months from date of recurrence (*p* < 0.00001). In the propensity score matched cohort, the survival benefit remained – 38 vs 19 months (*p* = 0.00038). In fact, median survival from date of recurrence after secondary intervention was similar to the survival noted after index surgery among all colorectal patients in the HIPEC registry (38 vs 41 months). Furthermore, patients who underwent index and secondary CRS+ HIPEC had a median survival of 60 months from the index procedure compared to 36 months in patients receiving palliative treatment upon recurrence (Fig. S3). Sensitivity analyses corroborated these results with very little variation. Based on these findings, we suggest that secondary CRS + HIPEC should be considered for all patients with recurrent peritoneal disease in colorectal cancer after initial CRS + HIPEC by evaluation in an MDT. There should be some caution exercised as a secondary CRS + HIPEC should not risk greater morbidity than the index procedure. Thus, the MDT evaluation of the tumor burden and the overall biological behavior of the recurrence need to be assessed.

The inherent selection bias in our study is difficult to completely overcome. To make the groups more comparable, a propensity score matching was undertaken. The four variables used to propensity score match patients were the factors found relevant in a proforma decision-making analysis (Table 2). PCI at index CRS and HIPEC, disease-free interval, and absence of extra-peritoneal metastases are known from the literature to be of importance in deciding whether to recommend secondary treatment, which was also found in our study [[5], [6], [7], [8], [9], [10], [11], [12], [13]]. Somewhat unexpected, we found a sex difference in patients being offered secondary CRS + HIPEC. The distribution was even in the palliative group, but in the interventional group 2/3 were women (Table 2). One may speculate that surgical re-intervention is considered less difficult in the female pelvis and abdomen. Among all patients with a peritoneal recurrence after CRS + HIPEC (*n* = 208), women had a better prognosis, although not statistically significant in the propensity score matched group (*n* = 74), Table 2, Table 4. It may be that women's tumor biology, for unknown reasons, is more favorable and that this might be amplified in the relapse setting with longer disease-free intervals. In summary of the decision-making analysis, it appears that patients being offered secondary CRS more often were female, of lower age (although not prognostically significant in the analysis), had lower PCI at index CRS, also lower PCI at secondary CRS compared to their own index PCI, had longer disease-free interval > 12 months, and more often isolated peritoneal recurrence (less oligometastatic).

The PSM model was successful in finding suitable matching pairs for 88 % (37/42) of patients receiving secondary CRS and HIPEC. We believe there is a reasonable comparability between the two PSM groups although there are baseline differences in age, tumor sidedness, neoadjuvant therapy, and operating time (Table 3). We have addressed each of these issues in different ways. Age and tumor sidedness were not prognostic factors in either of the cohorts (Table 2, Table 4). Neoadjuvant therapy was assessed in a separate manner (data not shown) yielding no prognostic impact which is in line with a large recent analysis on neoadjuvant therapy [14]. Operating time was not evaluated due to collinearity with PCI. Furthermore, several sensitivity analyses were performed to evaluate the robustness of the propensity score matched survival results. Results were similar, which strengthens the conclusions. Although our propensity score matching was successful without too many unmatched cases (*n* = 5 or 12 %), there may be unidentified factors, such as co-morbidity and recurrent disease extent, that play an important role in the patient selection process, which is why a comparison with survival from the index procedure is necessary to mitigate this selection bias issue. As mentioned earlier, the second intervention appears to “reset” the survival outcome achieving almost the same median survival as the index procedure achieves (38 vs 41 months).

Overall survival in this study was superior to most previously published series, with or without comparison groups [5,6,[8], [9], [10], [11], [12], [13]]. Probably, the main reason for this is the recent advances in palliative chemotherapy for advanced colorectal cancer. To our knowledge, only one other study has compared secondary CRS + HIPEC to palliative chemotherapy for recurrent peritoneal disease in patients who previously have undergone CRS + HIPEC for colorectal cancer PM [7]. In that study, which included patients from 2004 to 2015, 8 % of patients previously treated with complete CRS + HIPEC had secondary treatment (34/414), similar to the 7 % rate in our cohort (42/575). Interestingly, OS in the interventional group was basically the same between the two studies (36 vs. 38 months), whereas survival in the palliative group was twice as high in our cohort (7.6 vs. 15 months), who received treatment between 2010 and 2021. Despite the apparent improvement in systemic chemotherapy over time, there was still a significant difference in outcome between patients selected for secondary CRS + HIPEC or palliative chemotherapy. A randomized trial will be difficult to perform (although not impossible with international efforts) and we therefore recommend at least considering patients with recurrent PM for eligibility of secondary CRS+ HIPEC at a dedicated peritoneal surface oncology MDT. Further research is needed to determine which patients may benefit the most from secondary CRS + HIPEC.

In reference to the external validity of this study, the national Swedish HIPEC registry may not contain all patients treated for peritoneal recurrence following CRS + HIPEC. For example, a small isolated peritoneal metastasis may be viewed as a local recurrence and removed in a non-HIPEC hospital without complete abdominal exploration or HIPEC.

When reviewing the literature on systemic chemotherapy treatment of peritoneal metastases, survival ranges from 17 to 25 months, which is similar to our results for the propensity score matched cohort (19 months) [4,[15], [16], [17]]. Unfortunately, the registry does not cover systemic chemotherapy treatment following the diagnosis of a peritoneal recurrence after CRS + HIPEC. Therefore, it is unknown how many of the matched patients in the palliative group received systemic chemotherapy, and with what drugs. This limitation means that the palliative group should be considered an intention-to-treat group, which is also the case with the interventional group, where four patients were open/close cases. Nonetheless, the patient selection process has been successful in keeping the rate of futile secondary surgery attempts low.

Besides the aforementioned limitations, it is important to note that despite all efforts to mitigate selection bias, this is necessarily still a factor to consider. Certain variables on the primary tumor characteristics including molecular subgroups were not available in the registry, likewise the disease extent on the peritoneum at the time of recurrence. The only variable available to somewhat mitigate this was the disease-free interval as a surrogate marker for disease biology in general. Fortunately, this variable was successfully match with both groups having 15 months mean time to recurrence (Table 3). We have tried to use the pallative group as a reference together with other comparisons to provide a whole view over the outcomes achievable with secondary CRS rather than a strict comparison with the palliative group.

In conclusion, considering the unlikeliness that a randomized trial will be performed in this clinical situation any time soon, well-conducted observational studies can provide evidence on the possible benefits of secondary CRS + HIPEC. Keeping the limitations of this study in mind, survival after secondary CRS + HIPEC for colorectal PM appears not to be inferior to that after index treatment. Furthermore, secondary CRS + HIPEC markedly increased survival compared to systemic chemotherapy in the literature [4,[15], [16], [17]]. Thus, secondary CRS and HIPEC is a reasonable treatment option for selected patients with recurrent PM of colorectal cancer.

The following are the supplementary data related to this article.

## Funding

The authors received no funding for this work.

## Ethical approval statement

This study was approved by the Ethical Review Authority - Dnr 2023-03743-01.

## CRediT authorship contribution statement

**Peter Harald Cashin:** Conceptualization, Data curation, Formal analysis, Investigation, Methodology, Project administration, Resources, Visualization, Writing – original draft, Writing – review & editing. **Dan Asplund:** Conceptualization, Data curation, Resources, Writing – review & editing. **Elinor Bexe Lindskog:** Conceptualization, Data curation, Validation, Writing – review & editing. **Lana Ghanipour:** Conceptualization, Data curation, Writing – review & editing. **Ingvar Syk:** Conceptualization, Data curation, Resources, Writing – review & editing. **Wilhelm Graf:** Conceptualization, Data curation, Resources, Writing – review & editing. **Per J. Nilsson:** Conceptualization, Data curation, Resources, Writing – review & editing. **Gabriella Jansson Palmer:** Conceptualization, Data curation, Project administration, Resources, Writing – review & editing.

## Declaration of competing interest

The authors have no relevant financial disclosures.
